# User Engagement and User Loyalty Under Different Online Healthcare Community Incentives: An Experimental Study

**DOI:** 10.3389/fpsyg.2022.903186

**Published:** 2022-04-29

**Authors:** Mingxing Shao, Xinjie Zhao, Yafang Li

**Affiliations:** ^1^International Business School, Beijing Foreign Studies University, Beijing, China; ^2^Department of Management, Information Systems, and Entrepreneurship, Carson College of Business, Washington State University, Pullman, WA, United States

**Keywords:** online healthcare community, online incentive levels, user engagement, user loyalty, user perceived value, self-health management value

## Abstract

The online healthcare community (OHC) has attained rapid development in recent years in which users are facilitated to exchange disease information and seek medical treatment. However, users’ motivation of participation in OHCs is still under investigation. Taking the perspective of user perceived value, this paper examined the impacts of different incentive levels including identity incentive, privilege incentive, and material incentive on user perceived value, user engagement, and user loyalty. To test the proposed hypotheses, the study adopted the methods of the between-subjects experiment and questionnaire. Based on the data analysis by ANOVA and structural equation model, the results show there are significant differences in the impacts of different incentive levels on users’ perceived value. Most of the incentive measures exert significant effects on simple user hedonic value and community identity value. Accordingly, the research findings suggest that affective support value and self-health management value demonstrate more importance for user engagement and user loyalty. Therefore, OHCs should try to improve users’ affective support value and self-health management value which are the ultimate aims of the OHCs. Our study sheds some light on profoundly understanding the design of incentive mechanism of OHC and contributes to the research of OHC services.

## Introduction

Facilitated by the increasingly pervasive development of social media platforms, like blogs and forums, the online healthcare community (OHC) has become an important channel for users to exchange disease information and seek medical treatment. With advancement of information technology, more approaches are available for individuals to acquire knowledge on diseases and treatments (Li et al., 2021). People use social media to obtain healthcare information and exchange experiences of their medical treatment wherein social media enable people to manage more effectively their own health to a certain extent (Khurana et al., 2019). Previous research shows that social media has changed the interactive ways between doctors and patients, creating new medical treatment experiences (Liu et al., 2020). People may enjoy emotional support while acquiring health-related information from social media. They prefer to use the OHC not just for seeking health-related information or problem-solving, but also for viewing the community as a land to meet others and gain spiritual sustenance, such as emotional support, friendship, and sense of belonging (Fichman et al., 2011). It is reported that there are 100 million registered users and more than 15 million active users of OHC platforms in China. In China, active OHC platforms include HaoDaifu, ChunYu Doctor, etc. Globally, there are also a large number of active OHC platforms, such as PatientsLikeMe. Evidence also indicates that OHCs play a valuable role in patients’ problem-solving in China, especially after the outbreak of COVID-19.

Whether OHCs can create value depends on the number of participants and the content generated. The more users continue to participate, the more valuable the platform is. OHCs are supposed to provide expected services to attract potential users and promote users’ continuous participation and interactions. However, the effectiveness of OHCs is exponentially diluted by the users’ non-active participation and even withdrawal. It has been universally recognized that establishing and maintaining a high level of user engagement and user loyalty has become a major challenge for the effective operations of OHCs (Glasgow et al., 2011). One of the roadmaps the OHC service providers adopt to resolve this dilemma is to design various types of effective incentives to promote users’ willingness to participate.

Based on the previous literature, online incentives were mainly classified into two domains: intrinsic incentives and extrinsic incentives (Khern-Am-Nuai et al., 2018; Vilnai-Yavetz and Levina, 2018). Intrinsic incentives refer to incentives sourcing from the individual himself/herself, rather from external financial rewards or recognition. Intrinsic incentives derive from the self-satisfaction by taking the corresponding actions (Khan, 2017). On the other hand, external incentives refer to the incentives sourcing out of the individual himself/herself, such as recognition, products/service, and financial benefits. These rewards provide satisfaction and pleasure, which the individuals’ action itself may not contain (Bowles and Polania-Reyes, 2012).

In view of the importance of user engagement and loyalty, scholars have conducted a lot of research based on different scenarios, including brand engagement in offline consumption, user engagement in online brand community, and user engagement in social media (Claussen et al., 2013; Hollebeek et al., 2014). However, at present, there are few studies on user engagement and loyalty in OHC scenario, especially the studies on user loyalty of OHC. As mentioned above, online user engagement and user loyalty are crucial factors to invigorate online platforms, especially OHCs. Therefore, it is very necessary to carry out sufficient research on OHC user engagement and user loyalty. Moreover, in the existing research on online user engagement and user loyalty, the vast majority of literature accommodated user experience and user perceived value as antecedents, studied how user experience or user perceived value affects subsequent user behavior, and neglected how to improve online user engagement and loyalty from the perspective of design science. In particular, the research on user engagement and loyalty from the perspective of incentive measures of platform design is insufficient. Considering the limitations and gaps of existing research, this paper attempts to study the following questions:

(1) Are there differences in user perceived value under different OHC incentives?
(2) What are the relationships between OHC incentive levels and user perceived value?
(3) Whether and how user perceived value affects user engagement and user loyalty? If so, what types of user perceived value can?
(4) What are the relationships between user engagement and user loyalty?

To answer the above research questions, this study proposed relevant hypotheses based on extant literature and related theories. And the study established a virtual health community with different incentive intensities based on a Tencent App named Interest Tribes. Data were collected from a survey delivered to 105 participants in the experiment, and ANOVA and structural equation model were employed to verify the theoretical model and hypotheses.

Following this first section of the introduction, the rest of this paper is organized as follows. “Literature Review” is about literature review of incentive, user engagement, and user loyalty in OHC. “Conceptual Framework and Research Hypotheses” illustrates the theoretical model of the effect of incentives on user engagement and user loyalty in OHC. Thereafter, “Research Methodology” introduces research method, experiment design and procedure, and data collection. Following that, data analysis is conducted in “Data Analysis”. Research results are then discussed in “Discussion”. In the end, theoretical and practical implications of this paper, as well as future research, are concluded in “Conclusion, Limitation and Future Research”.

## Literature Review

### Online Healthcare Community

Online communities applying social media technologies, tools, and assistance, such as Facebook, Wechat, Youtube, Wikipedia, have become exponentially popular all over the world. With the widespread adoption of social media in various fields, people have recognized the enabling role of social media to reach a wide audience, and they largely started and enhanced users’ online interactions in health information and healthcare services (Eijk et al., 2013).

An OHC is a platform assembling different medical organizations, doctors, and patients together. On the OHCs, users can conveniently exchange and share medical experiences and knowledge with others in a timely manner. More specifically, individuals can consult with experts, learn from other patients, share their experiences with others, and gain useful health knowledge (Zhang et al., 2017). In this vein, OHCs have transformed the traditional medical services to the “patient-center care” pattern. More importantly, the OHCs provide convenient approaches for patients to seek health information, build relationships among users, provide and access social support (Alali, 2016). Facilitated by OHCs, patients can become active participants in their health management and improve their self-health management level (Amann and Rubinelli, 2017).

However, different from social media average users who meet online for common interests and hobbies, users use OHCs mainly to search for information and knowledge about certain physical or mental diseases. After their demands have been satisfied, they may discontinue to login in OHCs again. Therefore, the online health services community is supposed to provide a set of specialized design features, including personalized information, interactivity, content quality, and user friendliness to increase community cohesion and participation (Zheng et al., 2013; Ho et al., 2014).

Communities, especially commercial communities, are supposed to develop an effective business model by increasing user engagement and continuous engagement, namely user loyalty. In order to encourage users to be actively and continuously involved in online community interaction, it is worthwhile to further investigate how OHC service providers design appropriate incentives that well match users’ value expectations.

### User Engagement and User Loyalty of Online Communities

User engagement embodies interactive consumer experiences where ICTs such as social media act as tools that can enable and facilitate these experiences. The level of consumer engagement is reflection of calculative and affective commitment to an active relationship with a brand or its online community (Claussen et al., 2013).

Hollebeek et al. (2014) firstly focused on user engagement in social media which contains three facets covering cognitive processing, affection, and activation. Dessart et al. (2015) also contended that user engagement can be leveled with enjoyment/enthusiasm, attention/absorption, and learning/endorsing. Harwood and Garry (2015) took another perspective and argued that user engagement should include engagement mechanism, engagement emotions, and engagement behaviors. Accordingly, Verhagen et al. (2015) explicated that users participate in online community in the forms of accessing to knowledge, providing feedback, getting social ID and social ties, obtaining peer recognition as a means of self-expression and altruism. They also concluded that user engagement can produce cognitive benefits, social integrative benefits, personal integrative benefits, and hedonic benefits. Bitrian et al. (2021) concluded that user engagement leads to greater intention to use, disseminate WOM about, and to positively rate.

Compared with user engagement, user loyalty exerts greater and more profound impacts on online communities. User loyalty refers to the user’s preference and attitude toward a brand and their continuous repeated purchase behaviors (Jacoby and kyner, 1973). At present, it is generally believed that user loyalty is reflected in two aspects: behavior loyalty and attitude loyalty. In the current study, they are collectively referred to as user loyalty. As a typical online community, developing and maintaining the loyalty of OHC users can effectively increase users’ intention to revisit and help the website transform users at a low cost. In this regard, possession of a large number of loyal users in is a prerequisite for the online community to survive and function efficiently. Given the importance of user engagement and loyalty, scholars have conducted numerous studies from various academic disciplines, including consumer brand engagement, consumer engagement on social media, or online brand communities. However, insufficient research has ever endeavored to investigate user engagement from the perspective of incentive measures designing by the platform. In this paper, we applied incentive measures as the initiators of user engagement in the social media platform.

### Incentives Designing by Social Media Platforms

Studies have shown that users’ appropriate incentives will motivate them to participate in social media activities, including seeking information, sharing experience, offering assistance (Khan, 2017), contributing knowledge (Langtao et al., 2022), and offering online referrals (Jung et al., 2021). Khern-Am-Nuai et al. (2018) further argued that appropriate incentive and encouragement is the best way to maintain the loyalty of users, and the specifically differentiated targets for setting up incentive systems in various communities are slightly distinct. In this vein, how to motivate and maintain users’ engagement and loyalty in an online community by certain incentive mechanism design becomes a top concern (Khern-Am-Nuai et al., 2018).

Prior literature categorized incentives into intrinsic incentives and extrinsic incentives (Khern-Am-Nuai et al., 2018; Vilnai-Yavetz and Levina, 2018). The intrinsic incentives refer to the tactics that can lead to people’s psychological feelings in competence of doing some activities, such as privilege, honor, and right (Fu et al., 2017; Vilnai-Yavetz and Levina, 2018). By contrast, the extrinsic incentives are pertaining to the tactics that can provide material rewards, such as money, products, free services (Bowles and Polania-Reyes, 2012; Khern-Am-Nuai et al., 2018). Wirtz et al. (2013) concluded that there are three types of drivers for online communities which cover brand-related drivers, social-related drivers, and function-related drivers. To be specific, brand-related drivers include brand identification and brand’s symbolic function. Social-related drivers mainly refer to social benefits and social identity, while function-related drivers mainly contain function benefits, uncertainty avoidance, information quality, monetary and explicit normative incentive.

We explored the existing online Q&A communities and OHCs to observe what kinds of incentives those platforms are offering to their users. And we found that most OHCs are applying strategies of both intrinsic incentives and external incentives. For example, “Baidu knows” establishes points and grading system for the number of answers, and Weibo establishes points and hierarchy as a comparison between fans. External incentives have also been considered in existing OHCs, such as 39.com healthcare community, which always provides free common medicine or discounted medical service to online users.

### Perceived Values and OHCs Value Creation

Perceived values were firstly introduced in the field of marketing, describing the values that products provide to consumers. Anderson and Sullivan (1993) proposed that user perceived value is the users’ perceived utility of the product as a whole in terms of economic, social, service, and technical benefits. Further, scholars interpreted the connotations of users’ perceived value from different perspectives. For instance, Jillian and Geoffrey (2001) summarized perceived value into four categories, namely, emotional value, social value, functional value, and quality value. Holbrook (2006) identified four dimensions of consumer perceived value, functional, emotional, social, and altruistic. And Verhagen et al. (2015) argued that user perceived value should cover cognitive benefits, social integrative benefits, personal integrative benefits, and hedonic benefits.

In offline service occasions, user perceived value has been studied. Perceived values are considered to have positive effects on actual users’ attitudes and long-term usage behavior (Hu, 2021). Furthermore, many scholars studied online community behaviors as the consequence factors of user perceived value. For instance, Hollebeek et al. (2014) discovered that user value has a direct impact on user self-brand connection and usage intention. Accordingly, customer value demonstrates positive relationships with online repurchase behavior (Molinillo et al., 2020), eWOM (Wirtz et al., 2013), and online brand loyalty (Ray et al., 2012). However, there is insufficient literature researching the source of values for users’ engagement in OHC.

As far as OHC is concerned, most researchers put emphasis on motivation theory which contends that emotional support, social support, and information support are the main motivations driving users to participate in online healthcare communities (Hou and Shim, 2010; Kang and Lee, 2010; Zhang et al., 2017). These findings offer heavy hints for further investigation on incentive mechanism whereby users’ participation and engagement in OHCs is generated. To complement the extant literature, this paper categorized the perceived value into four dimensions, namely hedonic value (HV), community identity value (CIV), affective support value (ASV), and self-health management value (SHMV). In light of the significance of incentives, increasing research has been conducted about relevant effects of incentives, particularly on user engagement and user loyalty. To extend previous literature research findings, the current study investigated the mechanism wherein user perceived values function as roles mediating the relationships between incentives and user engagement/user loyalty.

**Figure 1:**
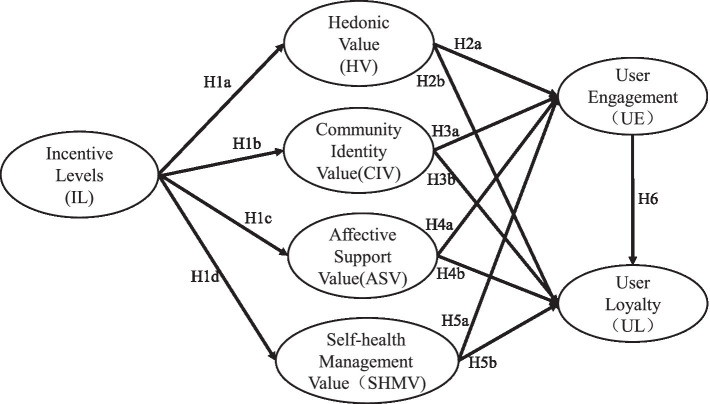
Theoretical model of incentive on user engagement and user loyalty in OHC.

Based on the above statement, the current research proposed the research framework (Figure 1), aiming to figure out the relationships between incentive levels, four dimensions of users’ perceived value, users’ engagement, and loyalty demonstrated in participating in OHCs.

## Conceptual Framework and Research Hypotheses

### Incentive Level and User Perceived Value

Appropriate design of the incentives is an effective way for community platform service providers to stimulate the participation of users. Likewise, online health community service providers take the same method to adopt many different incentives to attract users’ participation (Hulten, 2011). Prior literature has shown that categorized incentives and variant gradations of incentives demonstrate distinct effects on users’ engagement and loyalty of using social media (Dessart et al., 2015; Khern-Am-Nuai et al., 2018). Based on practical applications and previous literature, three user incentive mechanisms were designed with different intensity level: basic identity incentive, privilege incentive, and material incentive. Thereafter, the three types of incentives were combined into a three-tier incentive level system (ILS), which shows a progressive intensity influence. IL1 includes basic identification incentives. IL2 includes basic identification incentives and privilege incentives, and IL3 involves all the three types of incentives including basic identification incentives, privilege incentives, and material incentives.

#### Basic Identification Incentive

Applying basic identification incentives is the initial strategy taken by most social media platforms to attract and maintain platform users. This strategy manipulated in OHCs means that users will acquire corresponding levels of identity by accumulating participation points to reward their involvement in the OHCs. Attained levels of identity represent users’ reputation and their influence. Basic identification is always reflected in users’ activity points or usage duration which is designed to meet with people’s pursuit of reputation and identity (Marbach et al., 2019). According to the range of the integral value, users’ identity is graded into junior, intermediate, and senior, or even more detailed in most social media platforms. The strategy of basic identification adopted by social media platforms not only facilitates service providers to distinguish users of different quality, but also helps users to record their historical activities in the online community (Molinillo et al., 2020). Further, Fu et al. (2017) found that psychological motivation is an important antecedent affecting the knowledge sharing of users on Facebook. In this vein, the community should enable users to establish and maintain appropriate psychological incentives such as identity and privileges to encourage their engagement in virtual communities.

#### Privilege Incentive

In actuality, privilege incentives are regarded as a type of intrinsic incentives. These incentives are acquired by privileges which are well matched with the points users have accumulated during their participation in online communities (Khern-Am-Nuai et al., 2018). With relevant points acquired, users enjoy corresponding privileges such as special offers and policy permissions, including the number of post presence, post sharing, post deletion, and file download. Users who are more motivated to obtain information from online healthcare communities can increase incentives more effectively. As a result, their access to obtain wanted information or resources wins more facilitation (Jin and Huang, 2014). Evidence also suggests that individual users who actively share knowledge with online community members are offered with more privilege to access knowledge and this helps users improve their perceived knowledge gains and thus increase user participation (Khan, 2017).

#### Material Incentive

Material incentives apparently refer to external ones. Material incentives in OHC provide users with expected rewards in the forms of offline material rewards, including free diagnosis and treatment, free health products, and even some medicine. Evidence indicates that users’ continuous satisfaction on online communities basically derives from economic returns which stimulate users’ intention to be actively involved in the online communities (Bowles and Polania-Reyes, 2012). Online community should consider adopting different levels of incentive policies for users. Simple virtual incentives can help the community improve the users’ reputation and status in the community (Khern-Am-Nuai et al., 2018). However, for active participation users, the actual benefits, such as related gifts, coupon, and opportunities for some activities, are supposed to bring about more satisfaction (Vilnai-Yavetz and Levina, 2018). Many researchers argued that reward incentive system can lead to users’ more active participation in social media such as Facebook, Youtube, and other social Apps (Claussen et al., 2013).

When the platform provides users with a higher level of incentive combinations, users can perceive higher value as a response (Hassouneh and Brengman, 2014). Based on the perceived value theory, we defined users’ perceived value in OHC as the whole of users’ perceived utility, including economic, social, service, and technical benefits. In order to clarify how users’ perceived value in OHC functions, we divided the perceived value in OHC into four dimensions, namely HV, CIV, ASV, and SHMV.

Hedonic value means the joy and pleasure of using the various functions and services under different incentive mechanisms provided by the OHC; CIV refers to users’ sense of membership and belonging to the online health community, agreeing to its philosophy, design, and members; ASV interprets the emotional support and accompanying support that users obtain in the OHC; SHMC explains the users’ perceived ability and possibility of improving and managing their own health by using the OHC. Therefore, we proposed the following hypotheses:

*H1a*: Increasing incentive level (IL) has a positive impact on hedonic value (HV) in OHC.

*H1b*: Increasing incentive level (IL) has a positive impact on community identity value (CIV) in OHC.

*H1c*: Increasing incentive level (IL) has a positive impact on affective support value (ASV) in OHC.

*H1d*: Increasing incentive level (IL) has a positive impact on self-health management value (SHMV) in OHC.

### The Impact of Hedonic Value on User Engagement and User Loyalty

Hedonic value can also be interpreted as joys of use. Many studies have suggested that recreational motivation is one of the key factors driving users’ engagement in online communities. One of the starting points for the incentives provided by the community is to offer HV to users (Liu and Bakici, 2019). The higher the individual’s recreational qualities are, the higher enthusiasm for an activity users will demonstrate with better performance. By designing various incentive strategies, the OHCs fulfill the users’ demand of enjoyment, allowing users to perceive higher HV brought by the incentive mechanism. As a response, users’ engagement and user loyalty will be enhanced.

In this study, user engagement (UE) in OHCs refers to the combination of users’ participation mechanism, participation emotions, and participation behaviors (Harwood and Garry, 2015). Pertaining to participation behaviors, they mainly refer to acquiring health knowledge, providing feedback, obtaining social ties, and so on. User loyalty (UL) in this study refers to the commitment of a customer made to a particular brand or product which demonstrates potential power to influence users’ repurchase behaviors (Trenz et al., 2020), thereby affecting users’ commitment and tendency to continue to use certain OHC. Therefore, we proposed the following hypotheses:

*H2a*: Hedonic value (HV) has a positive impact on user engagement (UE) in OHC.

*H2b*: Hedonic value (HV) has a positive impact on user loyalty (UL) in OHC.

### The Impact of Community Identity Value on User Engagement and User Loyalty

Community identity value was originated from the concept of social identity. Tajfel (1978) coined and interpreted the connotations of social identity and believed that users can establish their social identity by recognizing their own membership in the group, building emotional connections to their membership, and making judgment on their own values. The social identity theory has been extended to the online virtual community and evolved into community identity. Moreover, the theory has been applied to explain the behaviors of virtual community users. Lim (2014) argued that when users have managed their social identity, they feel that their reputation and popularity has been increased with more value and respect attained from the online community. Users’ participation in online activities will create a sense of virtual community, including a sense of belonging and dependence. This emotional attachment is characterized by membership, influence, and immersion (Hou and Shim, 2010). When community members establish a more harmonious relationship and offer wide acknowledgment of enabling roles of each participant, users feel much easier to integrate into the community environment to exchange and share information, and achieve more overall social recognition (Amann and Rubinelli, 2017; Molinillo et al., 2020). Furthermore, users are more willing to participate in the interaction of the OHCs, which in turn enhance their loyalty and engagement. Therefore, the following hypotheses were made:

*H3a*: Community identity value (CIV) has a positive impact on user engagement (UE) in OHC.

*H3b*: Community identity value (CIV) has a positive impact on user loyalty (UL) in OHC.

### The Impact of Affective Support Value on User Engagement and User Loyalty

Facilitated by convenient internet access, users in most online communities are socially connected. The product designer of the community will try to create a user account linked with others, with which users can make friends with those who have the same or similar health demands in the community and thus a network of relationships has been developed. When users have more friends to communicate with, they encourage each other and obtain more ASV in the online health community (Alali, 2016; Marbach et al., 2019). At the same time, when users perceive more ASC, they will demonstrate more willingness to be actively engaged in OHCs, and thus their engagement and loyalty has been further cultivated (Molinillo et al., 2020). Therefore, users will show more responsiveness to the OHCs in a continual manner. Therefore, we proposed the following hypotheses:

*H4a*: Affective support value (ASV) has a positive impact on user engagement (UE) in OHC.

*H4b*: Affective support value (ASV) has a positive impact on user loyalty (UL) in OHC.

### The Impact of Self-Health Management Value on User Engagement and User Loyalty

Self-management is a self-regulating, dynamic, continuous, and interactive process that requires collaboration between family, community, and healthcare professionals. With the openness and anonymity of the OHC, users can consult on disease issues, search for health information, share knowledge and experience, make friends, and provide emotional support and even material support without any privacy concerns, thereby managing their own health more efficiently (Zhang et al., 2017). Given the fact that chronic is a complex, long-term, repetitious, and inconstant process, in which instructors cannot achieve comprehensive supervision of the patient’s physical condition in a sooner manner, in this regard, patient’s self-management is necessary and important (Eijk et al., 2013). Users always participate in online interaction for a particular purpose. The main goal of the OHC is to help users to improve their health condition (Glasgow et al., 2011; Dadgar and Joshi, 2018). If the perceived value of self-health management is high, users will show more willingness to participate in the OHC, and this will finally result in a higher frequency of online transaction. Therefore, we proposed the following hypotheses:

*H5a*: Self-health management value (SHMV) has a positive impact on user engagement (UE) in OHC.

*H5b*: Self-health management value (SHMV) has a positive impact on user loyalty (UL) in OHC.

### The Impact of User Engagement on User Loyalty

In order to maintain continuous user engagement, user loyalty is supposed to be well managed. User loyalty is accompanied with pleasant emotions created by customer experience and customer engagement. Researches show that customers with higher level of pleasure achieved in purchase experiences report more loyalty to the brands (Lemon and Wangenheim, 2009). Thus, we proposed the following hypothesis:

*H6*: User engagement (UE) has a positive impact on user loyalty (UL) in OHC.

Based on the above variable definitions and hypothesis analysis, this paper developed a three-tier incentive level system, abstracted the user perceived value into four dimensions, and proposed the research model to explicate the mechanism through which different incentive intensity levels exert impacts on the four dimensions of perceived value in OHC, thereby influencing user engagement and user loyalty. The proposed research model is shown in Figure 1.

## Research Methodology

The research methodology was composed of two parts. The first part was an experiential study conducted to test how the different incentive intensities produce different users’ perceived values. The second part was a survey study aiming to examine the influential mechanism of user perceived values on user engagement and user loyalty.

We established a virtual health community with different incentive intensity levels based on a Tencent App named Interest Tribes and selected samples at random to participate in the experiment. We collected data through questionnaires during the experiment and then employed ANOVA and structural equation model to verify the theoretical model and proposed hypotheses.

### Experiential Field and Experiment Design

These experiments are based on the Interest Tribe of Tencent QQ, which is a popular online community platform where people with the same interests gather together. In order to construct the OHC experimental scenario, we chose weight loss, a widely concerned health behavior, established 3 online weight loss communities, community *X*, *Y*, and *Z*, and set three different incentive intensities for them, respectively. According to the themes of weight loss that online users are concerned about, we designed three topics and relevant replies on exercise weight loss, diet weight loss, and spot weight loss, respectively, so as to create a relatively real online weight loss discussion and interactive environment for participants. Community *X* only offered basic identification incentives, and community *Y* used basic identification incentives and privilege incentives. Community *Z* adopted all incentive measures, including basic identification incentives, privilege incentives, and material incentives.

We designed the three communities by unifying the details of the community interface, experimental topics (constructing the community environment), and the previous style of the community’s posts to avoid potential impacts of other interference factors. The experiment design and user interface of the experimenter is shown in Appendix 1.

Before the formal experiment, we conducted focus group interview. The purpose of focus group interview is to determine the specific information parameters contained in the formal experiment, such as the detail measures that should be included in the three incentive intensities. A total of 4 in-depth participating users of online community, 5 in-depth participating users of OHC, and two professors in related fields participated in our focus group interview.

### Questionnaire Design

The items of the questionnaire were designed based on two standards. Firstly, the questionnaire items were chosen from the former literature and adapted necessarily in conformity with this research context. Secondly, they were designed in line with the definition of the variables to be measured.

We designed the variables involved in the research model and used the scales that have been verified by previous research and made appropriate modifications and adjustments according to the context of this study. This questionnaire adopted the 7-point Likert scale to do the rating according to the experimental content. 1 stands for strongly disagree, while 7 means strongly agree. The full version of the questionnaire survey is detailed in Appendix 2.

### Experiment Procedures

Before the formal experiment, we conducted pre-experiment. In total, 21 participants were selected for the pre-experiment. They were randomly divided into three groups with seven members in each group. Then they entered the corresponding community *X*, *Y*, and *Z* for simulation experiments. The results of the pre-experiment proved the rationality and feasibility of the experimental design.

In the formal experiment, we recruited a total of 105 participants. They were randomly and evenly divided into three groups with 35 people in each group. We named these three groups group I (identification incentive), group IP (identification incentive and privilege incentive), and group IPM (identification incentive, privilege incentive, and material incentive), respectively. Then we started the intergroup experiment. All members of group I got prepared for the community X experiment, group IP for the community Y experiment, and group IPM for the community Z experiment.

Each group was assigned with a researcher as the administrator who took charge of assigning distinct usernames, identities, and initial points to participants. Participants were informed of these three incentive mechanisms *via* experimental materials and community management explanations. The experiment was conducted with the following procedures:

Step 1: Before the experiment, the researchers briefly introduced OHC to all the participants. Participants joined the community according to the instruction of experimental materials, and at the same time, they completed the enrollment of usernames and identification. Participants were given 5 min to read instructions and policies of the community, especially the incentive mechanism.

Step 2: During the formal experiment, the participants had a free discussion on three topics created by the community administrator: exercise weight loss, diet weight loss, and local weight loss in the community, and the discussion lasted an hour. The main interactive behaviors of participants include posting new topics, posting reviews, deleting reviews, deleting topics, and so on. In order to establish a relatively real discussion and interactive environment, our design theme content was intercepted from the real online weight loss community.

Step 3: At the end of the experiment, we sent a questionnaire to each participant to fill in. Participants from three groups filled in the questionnaire which was scored on users’ four kinds of perceived values deriving from the experimental community, namely HV, CIV, ASV, SHMV, and the user engagement (UE) and user loyalty (UL).

Excluding the invalid questionnaires, we obtained a total of 100 valid samples, including 33 in group I, 32 in group IP, and 35 in group IPM. Each participant in the experiment received a gift worth about 50 yuan.

## Data Analysis

### Participants’ Demographic Characteristics

We recruited a total of 105 participants. They are all students from Beijing Foreign Studies University, mostly between the ages of 19 and 30. They are very familiar with various online communities and always actively participate in their preferred online communities. The participant’s characteristics are shown in Table 1.

**Table 1:** Participant’s characteristics.

Statistical variables	Option	Frequency	Percentage (%)
Gender	Male	38	37
	Female	67	63
Age	19–25	60	57
	26–30	45	43
Education level	Below undergraduate	3	2
	Undergraduate	67	58
	Graduate	61	37
	Doctor and above	5	3
Frequency of online health community use	At least once a day	21	13
	At least once a week	30	32
	1–3 times a month	30	32
	At least once every 3 months	25	15
	Once half a year or more	0	0
	Never	0	0

### Experimental Results: One-Way Between-Subjects ANOVA

This study adopted one-way ANOVA to test whether there are significant differences among the impacts of three intensity levels of incentive mechanism on the four dimensions of perceived value of users in the OHC. Four kinds of perceived value were measured using questionnaire data. Incentive levels were used as the grouping variable, wherein Group I was determined as 1 point, Group IP was determined as 2 points, and Group IPM was determined as 3 points. The researchers performed one-way between-subjects ANOVA, and the multiple comparison results are shown in Table 1.

For all the four aspects of perceived values, the difference among three incentive levels is significant concerning HV and CIV. However, the difference is not statistically different in terms of ASV and SHMV.

**Figure 2:**
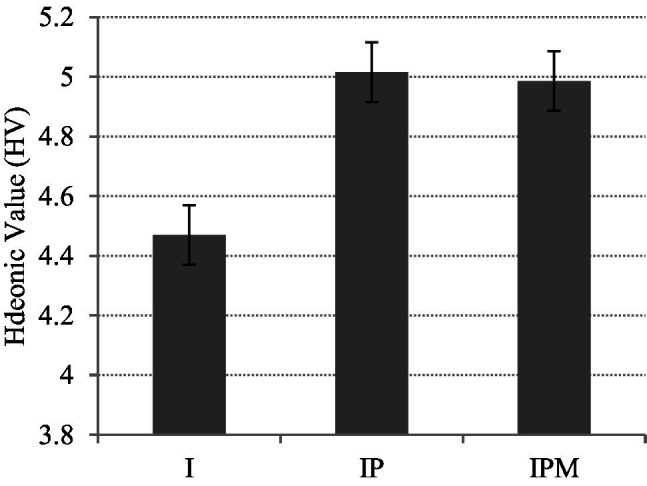
Hedonic value (HV) of three groups.

For HV, there are significant differences between Group I and Group IP (std. deviation = 2.18, *p* = 0.028), also between Group I and Group IPM (std. deviation = 2.06, *p* = 0.034) (see Figure 2). It shows that both privilege incentive and material incentive have substantial effects on users’ HV which can improve participants’ enjoyment level.

**Figure 3:**
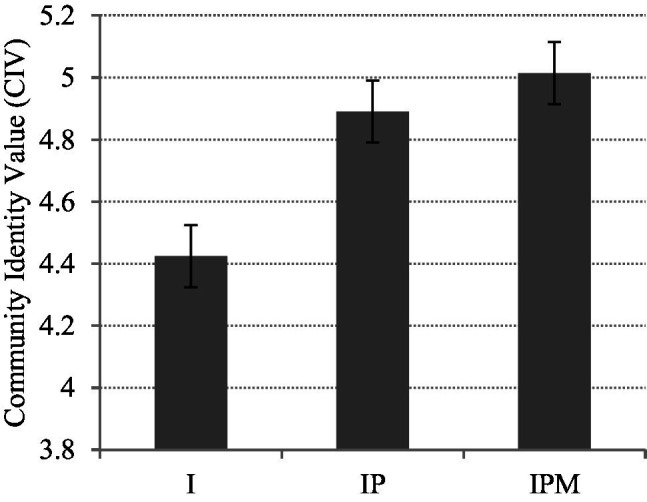
Community identity value (CIV) of three groups.

For CIV, there are significant differences existing between Group I and Group IPM which material incentive has a strong effect (std. deviation = 1.18, *p* = 0.030) (see Figure 3). However, privilege incentives demonstrate no significant effects on CIV.

For ASV, SHMV, no significant differences were found among three incentive intensity levels, Group I, Group IP, and Group IPM. These results show that the incentive measures provided by the platforms just display their effects on HV and CIV which are relatively lower level of perceived value. As far as the upper level of perceived value, like ASV and SHMV, these incentive measures do not show significant effects.

### Survey Results: Structure Equation Model

#### Factor Analysis

First of all, we took the exploratory factor analysis and principle component analysis and maximal rotation of variance. The KMO value of the scales was 0.697, which is greater than 0.5, and the Bartlett sphere test value was 1843.418 (*p* = 0.000), indicating that the data were suitable for factor analysis. Six factors were extracted and got a stable questionnaire structure. The total cumulative explanatory variance was 74.368%.

Based on the exploratory factor analysis, we performed the confirmation factor analysis. As shown in the analysis results (Table 2), all the factor loadings in confirmatory factor analysis of the measurement model exceeded 0.5 and were significant at *p* = 0.000.

**Table 2:** One-way between-subjects ANOVA—multiple comparisons.

Dependent variable	(I) Group	(J) Group	Std. deviation (I–J)	Std. error	Sig.	95% Confidence interval for mean	
						Lower bound	Upper bound
HV	I	IP	−2.1837^*^	0.98188	0.028	−4.1325	−0.2350
	IP	IPM	0.1196	0.96797	0.902	−1.8015	2.0408
	IPM	I	2.0641^*^	0.96028	0.034	0.1582	3.9700
CIV	I	IP	−0.9328	0.54883	0.092	−2.0220	0.1565
	IP	IPM	0.1196	0.96797	0.902	−1.8015	2.0408
	IPM	I	1.1801*	0.53676	0.030	0.1148	2.2454
ASV	I	IP	−1.7462	1.08699	0.111	−3.9036	0.4112
	IP	IPM	0.4536	1.07160	0.673	−1.6732	2.5804
	IPM	I	1.2926	1.06308	0.227	−0.8173	3.4026
SHMV	I	IP	−0.0492	0.84358	0.954	−1.7235	1.6250
	IP	IPM	−0.7179	0.83163	0.390	−2.3684	0.9327
	IPM	I	0.7671	0.82502	0.355	−0.8703	2.4045

* *The significance level of the mean difference is 0.05*.

#### Convergent Validity

Convergent validity is intended to measure whether there is internal consistency between the items in the questionnaire in order to indicate that the results are stable and reliable. There are usually three indicators, Cronbach’s alpha, construct reliability (CR), and average variance extracted (AVE). Cronbach’s alpha is generally considered that 0.65–0.7 stands for the minimum acceptable value. Construct reliability should exceed 0.8, and average variance extracted (AVE) by each construct should exceed the variance due to measurement error for the construct and generally should exceed 0.5.

Table 2 shows that all Cronbach’s alpha values were above 0.7, all CR values were above 0.8, and all AVE values were above 0.5.

#### Discriminant Validity

Discriminant validity assesses the extent to which a variable and its items differ from another variable and its items. There are two indicators, correlations between two variables and item loading of each variable. Correlations between two variables should be lower than the square root of the average variance shared by items within a construct, and item loading of each variable should be greater than the cross loading of any other variables (Fornell and Larcker, 1981).

In this study, as shown in Table 3, the square roots of AVE values of the factors on the diagonal lines in the table were larger than the corresponding correlation coefficients, indicating that the scale had good discriminant validity (Table 4).

**Table 3:** Reliability and convergence validity analysis.

Variable	Factor loadings	Cronbach’s α	Composite Reliability (CR)	Average Variance Extraction (AVE)
HV	0.859 0.859 0.846 0.735	0.845	0.896	0.683
CIV	0.896 0.901	0.762	0.894	0.808
ASV	0.802 0.838 0.895 0.868	0.874	0.913	0.725
SHMV	0.892 0.910 0.753	0.816	0.889	0.730
UE	0.748 0.801 0.816 0.635 0.596	0.772	0.845	0.525
UL	0.796 0.760 0.832 0.798 0.638	0.825	0.877	0.590

*HV, hedonic value; CIV, community identity value; ASV, affective support value; SHMV, self-health management value; UE, user engagement; UL, user loyalty*.

**Table 4:** Correlation coefficient matrix between square root of AVE value and factor.

	ASV	CIV	HV	SHMV	UE	UL
ASV	0.852					
CIV	0.620	0.899				
HV	0.635	0.617	0.827			
SHMV	0.348	0.361	0.267	0.854		
UE	0.591	0.495	0.550	0.413	0.724	
UL	0.703	0.681	0.638	0.509	0.660	0.768

*The value on the diagonal is the square root of AVE value*.

#### Structural Equation Modeling

**Figure 4:**
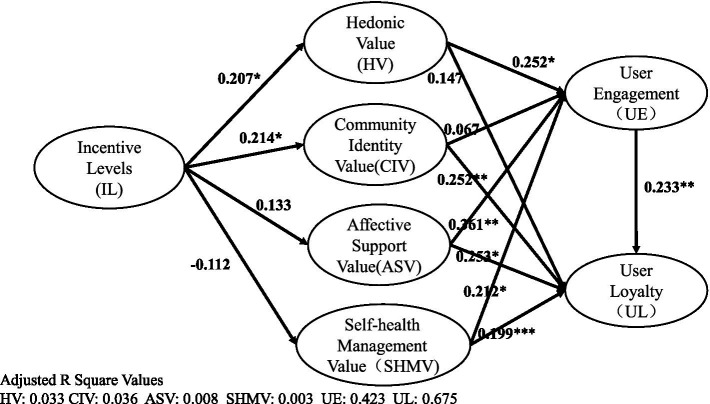
Results of the structural equation model. *p < 0.05; **p < 0.01; ***p < 0.001.

The structural model was further tested by Warp PLS which analyzes the model path relationships and validates the hypothesized variable relationships based on the results. *R*^2^ for user engagement (UE) is 0.446; *R*^2^ for user loyalty (UL) is 0.691. The model path coefficient results are shown in Figure 4, and the hypothesis test results are shown in Table 5.

**Table 5:** Hypothesis test of theoretical model.

	Hypotheses	Path coef. *β*	*T*-value	*P*-value	Results
H1a	IL → (+)HV	0.207	2.046	0.041	Supported
H1b	IL → (+)CIV	0.214	2.132	0.033	Supported
H1c	IL → (+)ASV	0.133	1.354	0.176	Not supported
H1d	IL → (+)SHMV	−0.112	1.108	0.268	Not supported
H2a	HV → (+)UE	0.252	2.278	0.023	Supported
H2b	HV → (+)UL	0.147	1.735	0.083	Not supported
H3a	CIV → (+)UE	0.067	0.505	0.613	Not supported
H3b	CIV → (+)UL	0.253	3.234	0.001	Supported
H4a	ASV → (+)UE	0.316	2.609	0.009	Supported
H4b	ASV → (+)UL	0.253	2.571	0.010	Supported
H5a	SHMV→(+)UE	0.212	2.306	0.022	Supported
H5b	SHMV→(+)UL	0.199	3.349	0.001	Supported
H6	UE → (+)UL	0.223	2.854	0.004	Supported

First, the findings indicated statistically significant relationships between incentive level (IL) and HV (*β* = 0.207, *p* = 0.041), CIV (*β* = 0.214, *p* = 0.033), respectively. Therefore, H1a and H1d are supported. However, the result indicated no statistically significant relationships between IL and CIV (*β* = 0.133, *p* = 0.176), ASV (*β* = −0.112, *p* = 0.268), as *p* values are much higher than 0.05. Therefore, H1c and H1d are not supported.

Secondly, the findings indicated statistically significant relationships between HV (*β* = 0.252, *p* = 0.023), ASV (*β* = 0.316, *p* = 0.009), SHMV (*β* = 0.212, *p* = 0.022), and user engagement (UE), respectively. Therefore, H2a, H4a, and H5a are verified. However, the finds showed no statistically significant relationship between CIV and user engagement (UE; *β* = 0.067, *p* = 0.613). Therefore, H3a is not supported.

Further, the results indicated statistically significant relationships between CIV (*β* = 0.253, *p* = 0.010), ASV (*β* = 0.199, *p* = 0.001), SHMV (*β* = 0.252, p = 0.001), and UL, respectively. Therefore, H3b, H4b, H5b are all verified. However, there is no statistically significant relationship between HV and UL (*β* = 0.147, *p* = 0.083). Therefore, H2b is not supported.

In addition, statistics indicates the significant relationship between UE and UL (*β* = 0.223, *p* = 0.004). Therefore, H6 is supported.

## Discussion

Closely focusing on the four research questions raised above, we began with this study by questioning are there differences in user perceived value under different OHC incentives. We proposed a theoretical model to investigate the impacts of three incentive levels (I, IP, and IPM) on the perceived values of OHC users (HV, CIV, ASV, and SHMV), and further on UE and UL.

The experimental result shows the incentive levels (IL) have different impacts on HV and CIV, but shows no significant effects on ASV and SHMV. Obviously, for HV and CIV, the adoption of higher level of incentives seems to work more efficiently. This also shows that no different levels of incentives demonstrate to be effective approaches influencing user self-health management awareness or their feelings of affective support in social media. These incentives can only affect the user perceived values with qualities of recreation and identity in virtual community, such as HV and CIV, but seldom have impact on ASV and SHMV, which will finally motivate users to change their health behaviors in reality.

The possible reason could be due to the fact that the cultivation of ASV and SHMV is a continuous and interactive process, which requires continuous and in-depth interaction between users and OHCs to transform the platform interaction into user perceived value acquisition. Especially in terms of SHMV, it is a process that requires users to continuously carry out health management and self-discipline in their offline life and then react to online perception. However, the experiment only lasted an hour. Therefore, even though the increase of IL may have potential effects on users’ ASV and SHMV, but the effects cannot display significantly within such a short period of time. For future research, further efforts examining the long-term effects of the IL on users’ ASV and SHMV deem to be necessary.

Structural equation model results prove again incentive levels (IL) have important impacts on HV and CIV, but not on ASV and SHMV. The research suggests that the analysis results made from both the structural equation model and one-way between-subjects ANOVA keep compatible.

From the analysis by the structural equation model, HV, ASV, and SHMV have significant and positive impacts on user engagement (UE). However, CIV has no significant and positive impacts on user engagement (UE). Further, CIV, ASV, and SHMV have significant and positive impacts on UL. However, HV has no statistically significant impacts on UL.

Users’ CIV comes from the sense of belonging and dependence on the community, which serves to strengthen their social identities in the online community and enhance their loyalty to the community. Due to the relatively limited time span for this experiment, participants have insufficient time to develop their effective community identity, which restricts their enthusiasm to participate in community activities to some extent. Therefore, from the statistical results of this experiment, users’ CIV has no significant impacts on UE. However, in the long run, CIV is very important for developing UL, which can significantly improve the tendency of users to participate in OHC.

Hedonic value can affect users’ willingness to engage in OHC as long as they can derive pleasure, fun, and entertainment from it. However, HV shows no statistically significant impacts on UL, which is a very interesting finding of this study. This indicates that HV does not naturally generate users’ loyalty to the OHC. This can be mainly explained by the fact that the utility derived from pleasure, fun, and entertainment only lasts a short period of time. Afterward, in the face of a large number of homogeneous platforms, users can easily shift their interests to other platforms. Therefore, simple perceived user value cannot maintain UE in a long run. To avoid users shifting to other OHCs, improving user’s CIV, ASV as well as SHMV deems to be necessary approaches. Online healthcare platforms should seek more measures to develop close and deep interactive relationships with users, which will finally contribute to the development of UL.

## Conclusion, Limitation, and Future Research

With the rapid development of OHCs in the recent years, the platforms designed various incentives in order to attract users. Both scientific research and corporate practices have shown that incentives are of great value to improve user perceived value in the OHCs. This paper studies the impacts of incentive measures on users’ perceived values, as well as the impacts on user engagement and user loyalty in the OHCs. The results provide implications on how to design effective incentive tools for OHC. It has far-reaching practical significance for promoting the development of OHCs. The results show most of the incentive measures only have effects on simple user values, such as HV and CIV. However, in a long run, ASV and SHMV demonstrate to be more important to develop UE and UL. Therefore, OHCs should try to improve users’ ASV and SHMV which are the ultimate targets of the OHCs. In this vein, future efforts could be allocated to research how to design a set of long-term incentives to profoundly enhance ASV and SHMV.

About the theoretical implications of this study, firstly, this study divides the incentives of online platform into three relatively independent and interrelated levels, and creatively combines them into a three-tier incentive level system. Secondly, this study takes user perceived value as the mechanism factor between incentive and user engagement and user loyalty, and explores the path of promoting user engagement and user loyalty through incentive design. Thirdly, this study further clarifies the concepts of user engagement and user loyalty, and reveals the relationships between them. Finally, this study builds a research model to explore relationships between incentive levels, four dimensions of users perceived value, user engagement, and loyalty demonstrated in participating in OHCs. These attempts are beneficial exploration and expansion of OHC mechanism study and user behavior study and provide a basis for relevant research in the future.

The practical implications of this study are to help OHC service providers design better platform incentive solutions to establish and maintain a high level of user engagement and user loyalty, so as to enhance the value of the OHC and promote the benign development of the whole industry. At the same time, better platform design can ultimately improve the user experience of the OHC, so that users can benefit more from the OHC, which is more conducive to users’ health management and health results.

Several limitations which may spawn future research ought to be noted. Firstly, the experimental subjects of the study were recruited in the university campus and most of them were college students. College students are the most active group in using various online communities and can be regarded as the most suitable representatives of online weight-loss community users. Nevertheless, there are still significant differences between college students and other groups considering age, education, income, and other factors which may result in the distinct differences in their perceived value, attitudes, and behavior in the OHC.

Applying the perspective of user perceived value, this paper employed the laboratory experiment and survey methods to study the theoretical model and test the corresponding hypotheses regarding the impacts of IL on user perceived values, UE, and UL in an OHC. Due to the limitations of experimental conditions, there are still certain differences between the designed online weight-loss community and the real OHCs. Therefore, for the future research, a longitudinal study or filed experiments could be considered to ensure high-quality data. Moreover, UE and UL may also be influenced by users’ personal attributes. Thus, user experience, user characteristics, and other individual features may be taken into account for future research to unveil a more comprehensive picture about UE and UL to the OHCs.

## Data Availability Statement

The raw data supporting the conclusions of this article will be made available by the authors, without undue reservation.

## Author Contributions

MS contributed to the study design and statistical analysis. XZ organized the experiments. YL wrote the first draft of the manuscript. All authors contributed to the article and approved the submitted version.

## Funding

This research was supported by the National Natural Science Foundation of China under the grants 71974018, 71573022, and 71874018, the G20 Research Center of BFSU under the grant G2020201003, and the Fundamental Research Funds for the Central Universities under the grant 2022JJ007.

## Conflict of Interest

The authors declare that the research was conducted in the absence of any commercial or financial relationships that could be construed as a potential conflict of interest.

## Publisher’s Note

All claims expressed in this article are solely those of the authors and do not necessarily represent those of their affiliated organizations, or those of the publisher, the editors and the reviewers. Any product that may be evaluated in this article, or claim that may be made by its manufacturer, is not guaranteed or endorsed by the publisher.
