# Supplementary material for: User Engagement and User Loyalty Under Different Online Healthcare Community Incentives: An Experimental Study
Source: Front Psychol. 2022 Apr 29;13:903186. doi: 10.3389/fpsyg.2022.903186 (PMC9100646; doi:10.3389/fpsyg.2022.903186)
Supplement: Supplementary file 1 [file Table_1.docx]

# Appendix 1 Experiment design

Community X: basic identification incentives

Community Y: basic identification incentives and privilege incentives

Community Z: identification incentives, privilege incentives and material incentives

Interface for Community X：


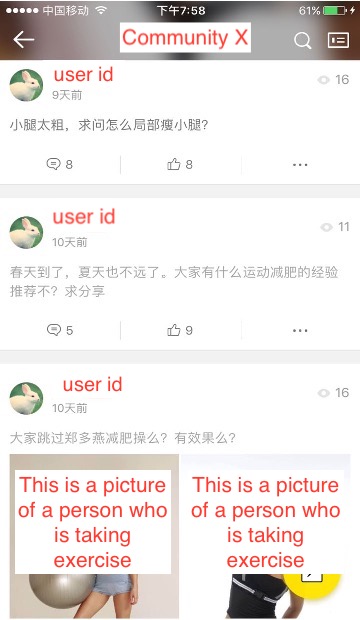


【Identification Incentives】

| Indentification | points | Policy for identification (several examples) |
| --- | --- | --- |
| Primary users | 0-299 | Login for 2 points；  review/like for 3 points；  start a topic for points；  get more than 20 comments for 10 points.  …… |
| Intermediate users | 300-500 |  |
| Senior users | >500 |  |

【Privilege Incentives】

| Privilege | Primary users | Intermediate users | Senior users |
| --- | --- | --- | --- |
| Browse the posts | √ | √ | √ |
| Start a topic | √ | √ | √ |
| Post a review | √ | √ | √ |
| Top topic | × | √ | √ |
| Any number of post within 24 hours | × | √ | √ |
| Delete the topic | × | × | √ |
| Delete the review | × | × | √ |
| …… |  |  |  |

【Material Incentives】

| Material incentives |
| --- |
| Free health products |
| Free health examination |
| Coupons,discounts, vouchers, gifts, etc. |
