# Supplementary material for: User Engagement and User Loyalty Under Different Online Healthcare Community Incentives: An Experimental Study
Source: Front Psychol. 2022 Apr 29;13:903186. doi: 10.3389/fpsyg.2022.903186 (PMC9100646; doi:10.3389/fpsyg.2022.903186)
Supplement: Supplementary file 2 [file Table_2.DOCX]

# Appendix 2 Questionnaire survey

| Community Identity Value | CIV1: I feel I am a member in the online community.  CIV2: I feel I am familiar with other members in the online community. | Aulia et al. (2016)  Hollebeek et al.(2014)  Sweeneya and Soutar(2001) |
| --- | --- | --- |
| Affective Support Value | ASV1: I feel positive when I use the online weight-loss community.  ASV2: I feel been cared for by other members in the online weight-loss community.  ASV3: I feel being understood by other members in the online weight-loss community.  ASV5: I feel be encouraged by other members in the online weight-loss community.  ASV4: The other members in the online weight-loss community are friendly. | Aulia et al. (2016)  Hollebeek et al.(2014)  Sweeneya and Soutar(2001) |
| Self-Health Management Value | SHMV1: I always learn from other users’ experience of diet and exercise in online weight-loss community.  SHMV2：It can encourage me to control my diet and improve my exercise in the weight-loss community.  SHMV3: The information in the weight-loss community always lead me to try new products and methods.  SHMV4: I want to show my weight-loss achievement in the online weight-loss community. | Self-design items |
| User Engagement | UE1：I always share information in the online community.  UE2：I always pay attention to the community’s activity.  UE3：I always response to other member’s topic.  UE4：I always interact with other members actively in the online community.  UE5：I am willing to spend time in the online community. | Hollebeek et al.(2014)  Verhagen, et al. (2015)  Khan(2017)  Sebastian et al. (2019) |
| User Loyalty | UL1：I like the online weight-loss community.  UL2：I always want to discuss some topics in the online community.  UL3：I can express my ideas freely in the online community.  UL4：I always get help in the online community.  UL5：I can get respect in the online community. | Hollebeek et al.(2014)  Verhagen, et al. (2015)  Khan(2017)  Sebastian et al. (2019) |
